# Kerion celsi caused by *Trichophyton tonsurans* in an adult^☆^

**DOI:** 10.1016/j.abd.2021.10.005

**Published:** 2022-07-15

**Authors:** Jacqueline Paulina Aguirre Sotelo, Víctor Manuel Tarango Martinez, Lucio Vera Cabrera

**Affiliations:** a Department of Dermatology, Instituto Dermatológico de Jalisco “Dr. José Barba Rubio”, Universidad de Guadalajara, Zapopan, Jalisco, Mexico; b Interdisciplinary Dermatological Research Laboratory, Hospital Universitario “Dr. José Eleuterio González”, Universidad Autónoma de Nuevo León, Nuevo León, Monterrey, Mexico

**Keywords:** Dermatomycoses, Tinea capitis, Trichophyton

## Abstract

Tinea capitis is an uncommon infection in adults, and predominantly affects women and the elderly with hormonal disorders and immunosuppression. Clinical features are often polymorphic and atypical. A kerion celsi case in an elderly female patient with type 2 diabetes mellitus and menopause is presented. The diagnosis was established by direct examination and the isolation of *Trichophyton tonsurans* in culture and typified by microculture. Treatment with prednisone and itraconazol was very successful. Recognizing the presentation of tinea capitis in adults will help clinicians to avoid delay in the diagnosis, awareness of the risk factors and provide early treatment to minimize sequelae of the disease.

## Introduction

Tinea Capitis (TC) is an uncommon infection in adults caused by dermatophytes invading hair follicles and shafts. In recent years, there has been an increase in incidence in this age group, especially in western countries. While the prevalence differs between countries. In Mexico, Medina et al. (2003) reported a rate of 2.9%. It is seen more frequently in women and the elderly. The involved factors include hormonal disorders like menopause, diabetes mellitus, systemic diseases, immunosuppression, use of corticosteroids, autoinoculation, and transmission from affected children. Since clinical features may be atypical they could delay diagnosis and pose a challenge for the clinician.^1^, ^2^, ^3^, ^4^, ^5^

## Case report

**Figure 1:**
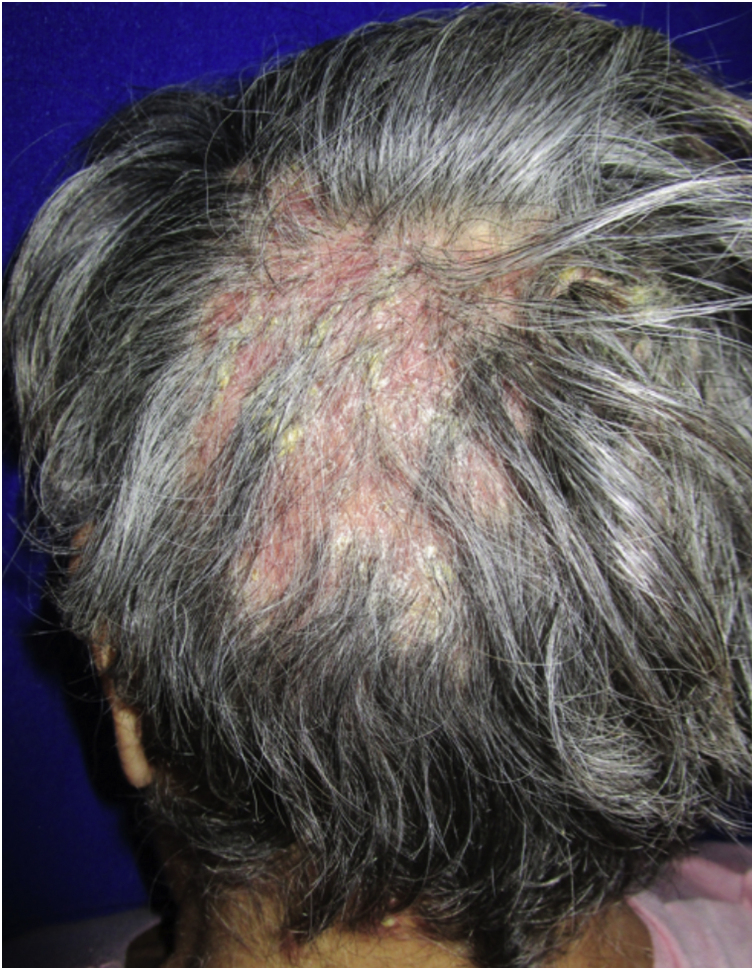
Erythematous pseudoalopecic plaques with pustules and yellow crusts involving 70% of the scalp.

**Figure 2:**
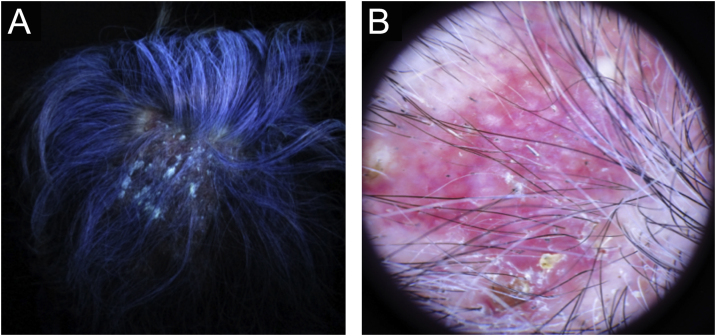
Ancillary diagnostic. (A), Wood’s lamp shows greenish fluorescence. (B), Trichoscopy features were comma hairs, broken hairs, black dots, perifollicular scaling, erythema, pustules and yellow crusts.

**Figure 3:**
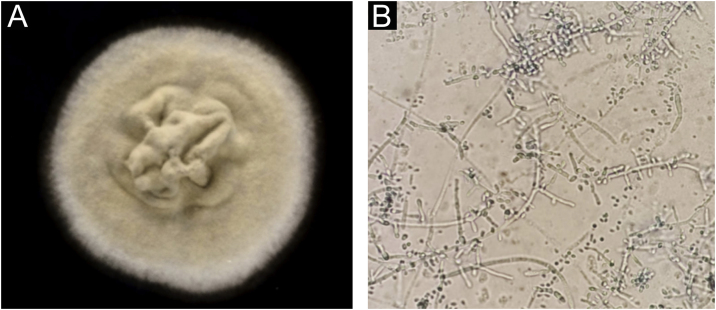
*Trichophyton tonsurans.* (A), Microbiological culture. (B), Thin and septate hyphae with chlamydoconidia and multiple conidia arranged in alternate ways and mirrored.

**Figure 4:**
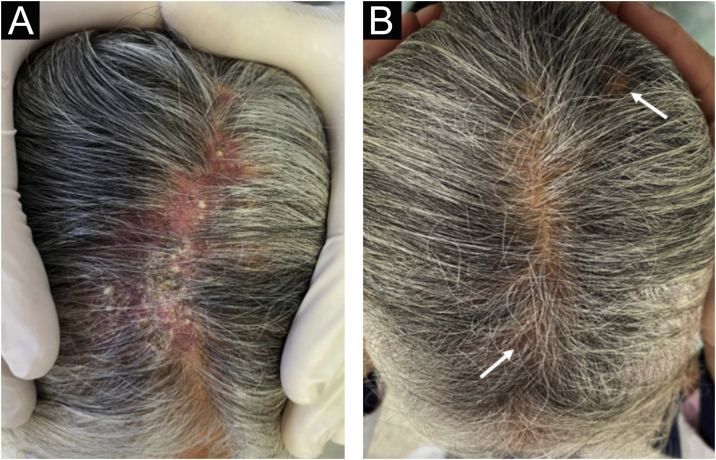
(A), View of the middle line of the scalp prior to treatment. (B), Hair regrowth post treatment with minimum scarring (arrow).

54-year-old female patient, with type 2 diabetes mellitus under treatment, no other clinical conditions or medication intake, who had no pets or children. Complained of lesions that began five months prior with scales, pustules, erythema, swollen and painful scalp with diffuse hair loss. The patient was medicated with oral antimicrobials and cream with betamethasone, gentamicin and clotrimazole for one month without success. The patient developed erythematous pseudoalopecic plaques with pustules and yellow crusts that involved 70% of the scalp (Fig. 1), tenderness and swelling were detected by palpation. The Wood’s light revealed a greenish fluorescence (Fig. 2), which lead to suspect in *M. canis* as an etiological agent, however, it could be a false positive from topical treatments and crust formation.^6^ At trichoscopy comma hairs, broken hairs, black dots, perifollicular scaling, erythema, pustules, and yellow crusts were found. The direct exam with 20% KOH showed an endothrix infection and the mycological culture grew a powdery, pale-buff and crateriform colony (Fig. 3). *Trichophyton tonsurans* were typified by microculture and their identity was confirmed by RNA operon sequence analysis using ITS1-ITS4 primers. Due to the inflammatory component, the authors started prednisone at 1 mg/kg per day for 4-weeks and itraconazole at 200 mg per day for 4 and a half months with almost full recovery of the hair and few remaining areas of scarring (Fig. 4).

## Discussion

The uncommonness of TC in adults is owing to the higher resistance to dermatophyte colonization due to the fungistatic properties of the scalp sebum, maturation of hair follicles, and the effectiveness of the immune system. It is thought that low estrogen levels in menopausal women, as well as in the elderly, cause the involution of sebaceous glands and decreased secretion of fungistatic sebum. Moreover, the changes in the thickness of the hair shaft may play a role in the susceptibility to being parasitized. In addition, the cases caused by *T. tonsurans* may be due to adults that are asymptomatic carriers of anthropophilic dermatophytes and develop TC only if immunodeficiencies or disruption of the scalp protective mechanisms occur. Social factors may also be involved, like being at a nursing facility, visiting the hairdresser, taking care of children, or having pets.^3^, ^7^, ^8^, ^9^

In the United States, Canada and Mexico, the most frequent etiologic agent is *T. tonsurans,* in Africa, India and Thailand, it is *T. violaceum,* and in some European, Asian and Latin-American countries prevails *M. canis.* Clinical features depend on the causative organism and the host’s immune response. Generally, TC doesn’t cause permanent hair loss, although it may be a result of a destructive inflammatory process of deep-seated TC. Differential diagnoses may include bacterial folliculitis, folliculitis decalvans, dissecting cellulitis, seborrheic dermatitis, aerate alopecia, psoriasis and scarring related to lupus erythematosus.^1^, ^2^, ^4^, ^9^^,^^10^ The patient was over fifty years old, had already reached menopause, suffered diabetes mellitus, and had previously used topical corticosteroids. All of these were risk factors for developing TC.

## Conclusion

Low clinical suspicion and inadequate empirical treatments delay the diagnosis and complicate the clinical course. Therefore, in the case of an adult or elderly patient with inflammatory skin changes on the scalp, even in absence of alopecic patches, the authors must rule out a dermatophyte infection as well as to inquire about predisposing factors. Provide early and accurate treatment to minimize sequelae of the disease.

## Financial support

None declared.

## Authors’ contributions

Jacqueline Paulina Aguirre Sotelo: Critical review of the literature, drafting and editing of the manuscript.

Víctor Manuel Tarango Martínez: Intellectual participation in the propaedeutic and therapeutic conduct of the studied cases; critical review of the manuscript and final approval of the final version of the manuscript.

Lucio Vera Cabrera: Molecular biology identification of the isolate; effective participation in research orientation; critical review of the manuscript.

## Conflicts of interest

None declared.
